# Disrupted tRNA Genes and tRNA Fragments: A Perspective on tRNA Gene Evolution

**DOI:** 10.3390/life5010321

**Published:** 2015-01-26

**Authors:** Akio Kanai

**Affiliations:** 1 Institute for Advanced Biosciences, Keio University, Tsuruoka 997-0017, Japan; E-Mail: akio@sfc.keio.ac.jp; Tel.: +81-235-29-0524; Fax: +82-235-29-0525; 2 Faculty of Environment and Information Studies, Keio University, Fujisawa 252-8520, Japan

**Keywords:** transfer RNA, split tRNA, tRNA fragment, molecular evolution

## Abstract

Transfer RNAs (tRNAs) are small non-coding RNAs with lengths of approximately 70–100 nt. They are directly involved in protein synthesis by carrying amino acids to the ribosome. In this sense, tRNAs are key molecules that connect the RNA world and the protein world. Thus, study of the evolution of tRNA molecules may reveal the processes that led to the establishment of the central dogma: genetic information flows from DNA to RNA to protein. Thanks to the development of DNA sequencers in this century, we have determined a huge number of nucleotide sequences from complete genomes as well as from transcriptomes in many species. Recent analyses of these large data sets have shown that particular tRNA genes, especially in Archaea, are disrupted in unique ways: some tRNA genes contain multiple introns and some are split genes. Even tRNA molecules themselves are fragmented post-transcriptionally in many species. These fragmented small RNAs are known as tRNA-derived fragments (tRFs). In this review, I summarize the progress of research into the disrupted tRNA genes and the tRFs, and propose a possible model for the molecular evolution of tRNAs based on the concept of the combination of fragmented tRNA halves.

## 1. Introduction

The three major RNAs involved in the flow of genetic information are messenger RNA (mRNA), ribosomal RNA (rRNA), and transfer RNA (tRNA). All these RNAs participate in the protein-synthesizing pathway in cells. The mRNAs essentially provide sequence information to specify amino acids, whereas the latter two RNAs, rRNA and tRNA, are directly involved in protein synthesis. tRNA is attached to the specific amino acid determined by the mRNA codon, whereas rRNA has a peptidyl transferase activity that is essential for peptide bond formation between aminoacylated tRNA substrates. In this context, tRNA has two distinct characteristics. It carries an anticodon corresponding to the mRNA codon and it binds to the corresponding amino acid in a reaction catalyzed by a specific aminoacyl-tRNA synthetase. In this sense, tRNA is a key bridging molecule between ribonucleotide information (RNA world) and peptide information (protein world). Therefore, tracing the evolution of tRNA molecules is likely to cast light on the processes that led to the establishment of the central dogma.

With the great progress in DNA sequencing technologies in the 21st century, especially next-generation sequencing techniques, huge numbers of complete and draft genomes have been registered in public databases. As of October 2014, for example, 3009 bacterial, 173 archaeal, and 21 eukaryotic complete genomes have been registered in the National Center for Biotechnology Information (NCBI) database (http://www.ncbi.nlm.nih.gov/genome/browse/). If we also take into account the draft genomes, the number swells by an order of magnitude. Since 2005, several groups, including our group, noticed that some tRNA genes that are essential for survival are absent from the genomes of particular Archaea and primitive Eukaryota. Because these tRNA genes are interrupted by non-canonical intron(s), or are even separated across two or three genes, these types of tRNA genes can rarely be discovered using conventional prediction software. Therefore, several groups developed new algorithms for computational prediction and found novel disrupted tRNA genes, including multiple-intron-containing tRNA genes [1], split tRNA genes [2,3,4,5], and permuted tRNA genes [4,5,6,7,8].

Both gene duplication and recombination are major driving forces in gene evolution, changing gene structures and functions. In the first half of this review, I summarize the accumulated observations of disrupted tRNA genes. Recent studies have showed that even mature tRNAs are processed to generate functional small tRNA fragments. In the second half of this review, I speculate how the combinations of the tRNA halves are important in generating the tRNA molecules seen today.

## 2. Intron-Containing tRNAs on the Domains of Life Tree

**Figure 1:**
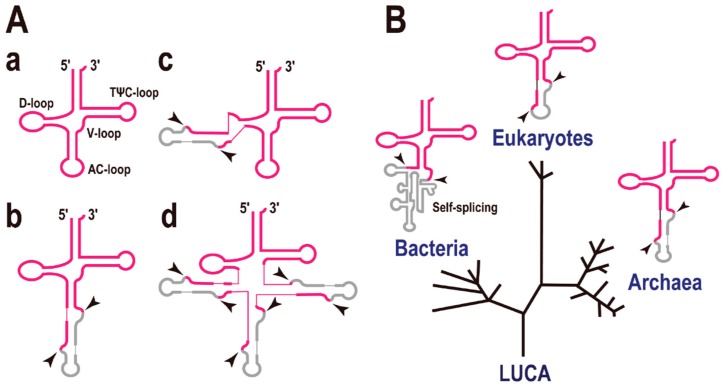
Intron-containing transfer RNAs (tRNAs). (**A**) Molecular structures of disrupted tRNAs. Exons are indicated in red and introns in grey. (**a**) Common tRNA. A description of each stem and loop is included; (**b**) tRNA containing a single intron at the canonical position, 37/38; (**c**) An example of a tRNA containing a single intron at a non-canonical position; (**d**) Example of a tRNA containing multiple introns (up to three introns) at various positions. The two arrowheads indicate the exon-intron boundaries cleaved by the splicing endonuclease. (**B**) The three domains of life and tRNAs containing a single intron. A phylogenetic tree representing the separation of the Bacteria, Eukaryotes, and Archaea. LUCA, last universal common ancestor. Most introns are located in the anticodon loop region of the tRNAs. Note that the secondary structures of the eukaryotic and archaeal tRNA introns are very similar and that the introns of both are processed by a specific enzyme, the tRNA splicing endonuclease. By contrast, the bacterial tRNA intron is a self-splicing-type intron.

The secondary structure of tRNA can be represented as a cloverleaf with four stems (Figure 1A-a). In some cases, tRNA genes are interrupted by intronic sequence(s) like eukaryotic protein-encoding genes. However, the introns in precursor tRNAs (pre-tRNAs) do not precisely resemble those in precursor mRNAs (pre-mRNAs). The eukaryotic pre-mRNA intron is recognized by base pairing to short RNA molecules such as U1, U2, U4, U5, and U6, whereas the pre-tRNA intron is recognized by a specific enzyme (complex), the tRNA splicing endonuclease [9,10]. Many of the pre-tRNA introns are found at a position adjacent to the anticodon, between nucleotides 37 and 38 of the precursor tRNA (37/38), known as the “canonical” position (Figure 1A-b). The tRNA intron forms a specific RNA secondary structure, the bulge-helix-bulge (BHB), with parts of the exon sequences [11], and this structure is the target of the tRNA splicing endonuclease (shown by arrowheads in Figure 1A) [12]. Using a systematic computational approach we found that some pre-tRNAs possess a single intron located at an unconventional site (Figure 1A-c), or even three introns (Figure 1A-d) in the Archaea, especially in the phylum Crenarchaeota. For example, pre-tRNA^Glu^(UUC) in *Pyrobaculum calidifontis* and pre-tRNA^Pro^(UGG) in *P. islandicum* both contain three introns [1]. However, the evolutionary view is that introns at non-canonical positions are rare, whereas introns at canonical positions (or in the anticodon loop region) are conserved in the three domains of life, Bacteria, Archaea, and Eukaryota (Figure 1B) [13].

Among the three domains of life, the mechanism of pre-tRNA splicing by endonucleases and their target BHB structures are conserved in both the Archaea and Eukaryota, whereas self-splicing catalytic introns (ribozymes), including the group I and group II introns, are present in the Bacteria [14,15]. Although there is no evidence for the sequence/mechanistic relationships between the ribozyme introns in Bacteria and the Archaea/Eukaryote systems, it is noteworthy that all these examples have an intron in the anticodon loop region. In other words, the introns located in the anticodon loop region seem to be evolutionarily ancient and may have existed at the beginning of life [16]. It is also suggested that many of tRNA introns located at non-canonical positions are “translocatable introns” [17] and may have appeared at a later stage of tRNA evolution. On the contrary, the main objection to this view is that many processing events and intron insertion events have occurred in the anticodon loop because the anticodon loop is the most exposed part of the tRNA molecule (structural/mechanistic reasons).

## 3. Split tRNAs and Tri-Split tRNAs

**Figure 2:**
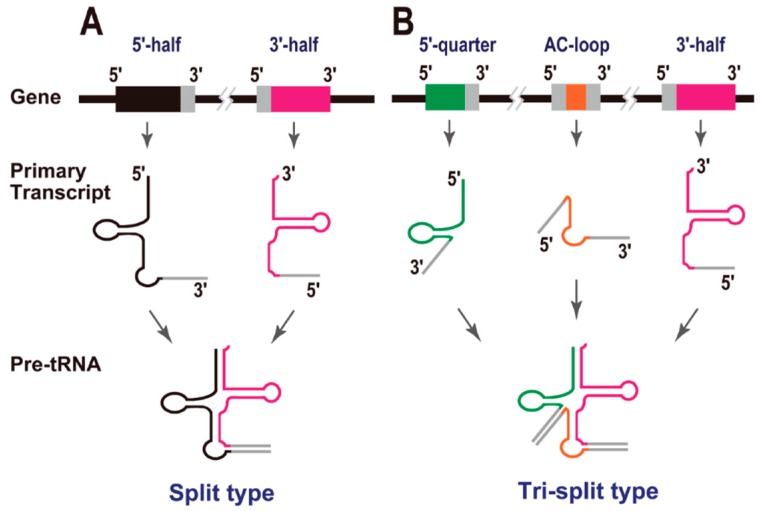
Split tRNAs. (**A**) Gene structure, primary transcripts, and precursors of split tRNAs. Exonic regions are colored in either black (5' half) or pink (3' half). Leader sequences are shown in grey. (**B**) Gene structures, primary transcripts, and precursors of tri-split tRNAs. Exonic regions are colored in either green (5' quarter), orange (anticodon loop), or pink (3' half). The secondary structures (BHB motifs) formed by the pairing of each leader sequence have been omitted from these cartoons for clarity.

In 2005, Randau *et al.* reported that the hyperthermophilic archaeon *Nanoarchaeum equitans* creates functional tRNAs from separate genes for their 5' and 3' halves (Figure 2A). This was the first report of “split tRNAs” [2], and a total of six tRNA genes (tRNA^iMet^[CAU], tRNA^His^[GUG], tRNA^Lys^[CUU], tRNA^Gln^[UUG], tRNA^Glu^[CUC], and tRNA^Glu^[UUC]) are generated by the combination of two tRNA halves [5]. It is noteworthy that two split tRNAs, tRNA^Glu^(CUC) and tRNA^Glu^(UUC), share the same 3' tRNA half-transcript. An RNA-seq study of the tRNA half-precursors in this organism supported the *trans* splicing of these tRNAs [18]. Because *N. equitans* is a parasite, with evidence of massive genomic reduction [19], it was unclear whether its tRNAs represented a form of tRNA that was unique to particular archaeal species or whether they were a later product of its genomic reduction. In 2009, we reported split tRNA genes [3] in a free-living organism, the hyperthermoacidophilic archaeon *Caldivirga maquilingensis*, which belongs to the deep-branching archaeal order Thermoproteales and was isolated from an acidic hot spring in the Philippines. In this case, four split tRNAs, tRNA^Gly^(CCC), tRNA^Glu^(UUC), tRNA^Ala^(CGC), and tRNA^Ala^(UGC), are generated by the combination of tRNA halves. Interestingly, two tRNAs, tRNA^Ala^(CGC) and tRNA^Ala^(UGC), share the same 5' tRNA half-transcript. Recently, split tRNAs have also been found in several different species of the Desulfurococcales branch of the Crenarchaeota: tRNA^Asp^(GUC) in *Aeropyrum pernix* and *Thermosphaera aggregans*, and tRNA^Lys^(CUU) in *Staphylothermus hellenicus* and *S. marinus* [4]. These observations suggest that split tRNA genes have spread sporadically across a major branch of the Archaea [20]. It should be mentioned that split tRNAs and intron-containing tRNAs share a common BHB motif around their intron/leader-exon boundaries, which can be cleaved by the same tRNA splicing endonuclease [3,21]. We have also demonstrated that the intervening nucleotide sequences of split tRNAs display high identity to the tRNA intron sequences located at the same positions in intron-containing tRNAs in related archaeal species [3]. These observations suggest an evolutionary relationship between these disrupted tRNAs.

During the analysis of the *C. maquilingensis* genome, we found that two mature tRNA^Gly^s, tRNA^Gly^(UCC) and tRNA^Gly^(GCC), are generated from three different transcripts and were designated “tri-split tRNAs” (Figure 2B). To construct mature tRNA^Gly^(UCC) and tRNA^Gly^(GCC), this archaeal species uses the same 3' half-transcript from the above-mentioned split tRNA^Gly^(CCC). However, the 5' half-regions of these tRNAs are separated into two different parts: the same 5' quarter and two alternative anticodon-loop transcripts. In other words, three tRNA^Gly^ species with synonymous codons (one split tRNA and two tri-split tRNAs) are created from a single constitutive 3' half-transcript and four alternatively switched transcripts [3].

## 4. tRNA-Derived Fragments (tRFs)

**Figure 3:**
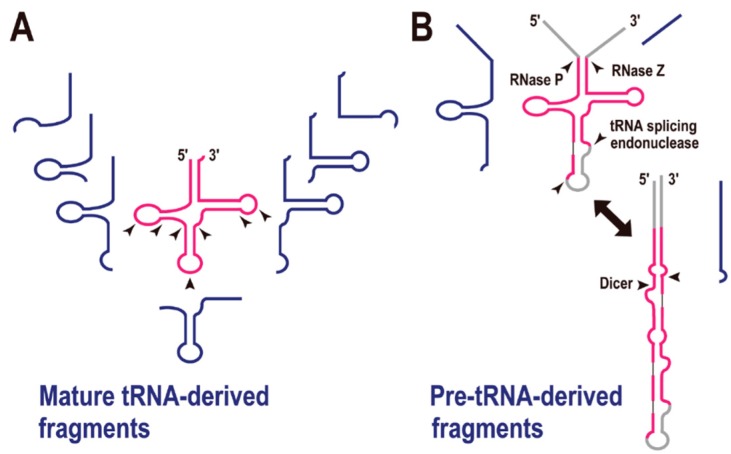
tRNA-derived fragments (tRFs). (**A**) Mature tRNA-derived fragments (shown in blue). The arrowheads indicate the possible cleavage sites by endoribonucleases. (**B**) Pre-tRNA-derived fragments (shown in blue). Enzymes required for pre-tRNA processing are shown with each arrowhead.

In the first decade of the 21st century, small non-coding RNAs, such as microRNA (miRNA) and small interfering RNA (siRNA), were recognized as major factors in gene regulation. A recent analysis of transcriptomes in all three domains of life has suggested that tRNA-derived fragments (tRFs) constitute a novel class of small regulatory RNAs. As reviewed by Raina and Ibba [21], there are many examples of tRFs (Figure 3A). In the initial stage of this research, the greatest concern was that these tRFs might simply be the degradation products of mature tRNAs. However, at least some of the tRFs appear to be biologically functional, based on the following observations: (i) in many cases, tRFs do not derive from abundant cellular tRNAs, and the numbers of tRFs do not correlate with the parental tRNA gene copy numbers; (ii) their fragmentation patterns are depended on their anticodons; (iii) the fragmentation patterns can change according to the developmental stage or cell conditions; and (iv) it is reported that some tRFs are bound to Argonaute/Piwi proteins, well-known components of the RNA-induced silencing complex [21,22].

The most frequently observed site of tRNA cleavage is within the anticodon loop, and tRNA genes are sometimes disrupted by an intron in this region. For instance, culture-condition-dependent or developmental-stage-dependent tRNA halves are observed in the fungus *Aspergillus fumigatus.* In this organism, the majority of tRNA 5' halves contain the anticodon sequence at their 3' ends, and the tRNA 3' halves commence immediately after the anticodon. The authors suggested that conditional tRNA depletion by their conversion to tRFs might be related to the downregulation of protein synthesis [23]. Developmentally regulated tRNA halves in the bacterium *Streptomyces coelicolor* [24] and stress-induced tRNA-halves in the yeast *Saccharomyces cerevisiae* have also been reported [25]. In the latter organism, the RNase T2 family member Rny1p is involved in tRNA cleavage following oxidative stress [26]. A deep-sequencing analysis of small RNA fractions showed that, in addition to the tRNA halves, further tRFs are cleaved at various nucleotide positions (Figure 3A) [27,28,29,30]. Moreover, our meta-transcriptomic analysis of microbes in an ocean-front deep sub-surface hot spring (*i.e.*, an environmental sample) was indicative of type-specific tRFs [31].

pre-tRNA is also the source of a tRF in mouse embryonic stem cells (Figure 3B) [32]. The authors suggested that an alternative secondary structure to the tRNA cloverleaf is a substrate for Dicer, the key enzyme in the pre-miRNA processing pathway. In fact, the generation of many tRFs is reported to be Dicer-dependent [33], although the exact secondary structures of the substrate tRNAs remain to be determined. Recently, a meta-analysis of tRFs revealed that they are associated with the Argonaute family proteins [22], which are also crucial enzymes in RNA silencing regulation. All these reports strongly suggest that some tRFs are involved in gene silencing regulation, in the same way as miRNAs. Thus, the recent tRFs have their own specific functions (including in RNA silencing), other than as adapter molecules in translation.

## 5. Possible Evolutionary Scenarios for tRNA Molecule

**Figure 4:**
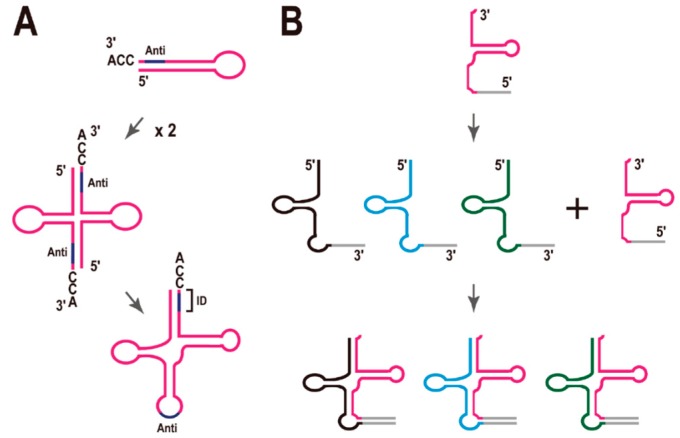
Models for the molecular evolution of tRNA. (**A**) Model of the origin of the tRNA molecule proposed by Di Giulio [34]. Anti, anticodon; ID, the region defining the identity of the tRNA. (**B**) Model of the early evolution of tRNA molecules inspired by the analysis of split tRNAs. See text for details.

Di Giulio hypothesized that the ancestral tRNA was encoded by two separate minigenes, which later fused to encode the modern tRNAs (Figure 4A) [34]. In fact, there are reports that the complete cloverleaf structure of tRNA is not necessary for tRNA function. For example, (i) a mini-helix RNA was efficiently aminoacylated by *Escherichia coli* leucyl-tRNA synthetase [35] and (ii) a top-half tRNA mini-helix is a good substrate for the eubacterial CCA-adding enzyme [36]. It was also recently reported that a ribozyme of only 5 nt effectively generated aminoacyl-RNA (self-aminoacylation) [37]. However, it is also true that there are no such short functional tRNAs in the current species.

In this review, I first summarized the characteristics and diversity of disrupted tRNA genes, focusing in particular on the archaeal genomes. Because the disrupted tRNA genes, including tRNA genes containing multiple introns and split tRNA genes, are predominantly observed in the archaeal order Thermoproteales (Crenarchaeota), one of the earliest branches within the kingdom, the question arises: Do disrupted genes reflect an ancestral character state?

As mentioned in Section 2, tRNA introns located in the anticodon loop region appear to be evolutionarily ancient, whereas tRNA introns located at non-canonical positions are translocatable, and these introns may have been acquired at a later stage of tRNA evolution. These observations suggest that tRNA introns have different origins, depending on their sites of insertion. There are also evolutionary relationships between the tRNA introns and the leader sequences of the split tRNAs [3,20]. The structural characteristics of split tRNAs carrying leader sequences in the anticodon loop region (see Figure 2A) are consistent with the hypothesis of Di Giulio. Here, Tanaka and Kikuchi reported that most tRNA sequences have vestiges of double hairpin folding [38]. In that report, each hairpin corresponded to either the 5' or 3' tRNA half. In other words, either the 5' or 3' half of the tRNA forms each alternative secondary structure with a mini-helix RNA, which is important for some tRNA functions, as mentioned above. Di Giulio’s model is supported by this finding. Previously, we mimicked a split tRNA by artificially separating the tRNA sequences of seven primitive archaeal species at the anticodon loop, and analyzed the sequence similarities and diversity of the 5' and 3' tRNA halves [39]. A network analysis revealed topological differences between the tRNA halves, suggesting different evolutionary backgrounds for the 5' and 3' tRNA halves, although they are short fragments of a single molecule. Furthermore, there was a correlation between the different combinations of representative 5' and 3' halves and the amino-acid type encoded according to the genetic code [39]. This concept is also supported by the alternative isoforms of the split tRNAs. To date, all isoforms can be created from one constitutive 5' or 3' half-transcript and an alternative half-transcript. Moreover, all alternative isoforms possess certain synonymous anticodons in each species. Therefore, I suggest that the combination of 5' and 3' half-transcript has not been random during evolution.

Based on these considerations, I propose a modified model for tRNA evolution (Figure 4B). Because the network analysis showed that the nucleotide sequences of the 3' tRNA halves are more conserved than those of 5' tRNA halves during archaeal evolution [39], and the end of the 3' tRNA halves (the CCA sequence) is essential for the addition of the amino acid residue to the RNA, I speculate that the 3' tRNA halves appeared first in the very early evolution of the tRNA molecule (perhaps during chemical evolution), and that asymmetric combinations with different 5' tRNA halves could have generated a variety of tRNA species at a later stage of evolution (Figure 4B), although I cannot posit the exact time point of this combination during the evolution of the molecule. LUCA may have had cloverleaf tRNAs like the current tRNAs. However, even so, it is possible that combinations of different 5' and 3' tRNA halves generated a variety of tRNA species. It must be mentioned that there is no example of a translocatable intron [17] or split tRNA [4] in the class II tRNAs, such as tRNA^Leu^ or tRNA^Ser^, each of which has an additional stem and loop structure—the long variable arm (V-arm). This finding supports the previous suggestion that class II tRNAs evolved relatively recently [40].

Randau and Soll have also suggested that disrupted tRNA genes arose by gene separation during the process of genomic rearrangement, and have since been maintained as a protective mechanism against the integration of mobile genetic elements (e.g., conjugative plasmids and/or viruses) because non-intronic tRNA genes are ideal targets for site-specific recombination [41]. It has also been suggested that at least some split tRNAs were generated from intron-containing tRNAs at a later stage of tRNA evolution [4,17]. Recently, a composite genome of the deep-branching archaeon *Caldiarchaeum subterraneum* was reconstructed from a community genomic library [42]. Based on a sequence analysis of the community library, we proposed that the gain of the tRNA intron and the scattering of the tRNA fragments took place within a short time-frame via the integration and recombination of a mobile genetic element [43]. Therefore, it is possible that the same mechanism led to the fragmentation of the tRNA genes, even during the later stages of molecular evolution, generating many new introns at non-canonical positions within tRNAs. However, it seems more likely that tRNA fragments and split tRNAs were present during the early evolution of tRNA molecules because there is no obvious route for generating a new gene disruption system at a later stage of molecular evolution. Further analysis and new findings are required to substantiate this view.

## 6. Conclusions

(a) Asymmetric combination of 5' and 3' tRNA halves may have generated the diversity of tRNA molecules.
(b) Even recently, tRNA genes have divided into 2–3 segments, and tRNA molecules themselves can fragment post-transcriptionally.
